# Efficacy and safety of etanercept biosimilar rhTNFR-Fc in Chinese patients with juvenile idiopathic arthritis: An open-label multicenter observational study

**DOI:** 10.3389/fped.2022.992932

**Published:** 2022-10-10

**Authors:** Xuefeng Xu, Xiaohui Liu, Wenjie Zheng, Jihong Xiao, Xiaozhong Li, Ling Wu, Lixia Zou, Qian Ouyang, Yaoyao Shangguan, Kezhao Lin, Xiaomei Dai, Yuanling Chen, Yiping Xu, Jianqiang Wu, Meiping Lu

**Affiliations:** ^1^ Department of Rheumatology Immunology / Allergy, The Children's Hospital, Zhejiang University School of Medicine, National Clinical Research Center for Child Health, Hangzhou, China; ^2^ Department of Rheumatology and Immunology, Jiangxi Provincial Children's Hospital, Nanchang, China; ^3^ Department of Paediatric Rheumatology, The Second Affiliated Hospital and Yuying Children's Hospital of Wenzhou Medical University, Wenzhou, China; ^4^ Department of Rheumatology and Immunology, Xiamen University Affiliated First Hospital, Xiamen, China; ^5^ Department of Rheumatology and Immunology, Soochow University Children's Hospital, Suzhou, China; ^6^ Department of Rheumatology and Immunology, Ningbo Women and Children's Hospital, Ningbo, China

**Keywords:** juvenile idiopathic arthritis, etanercept biosimilar, disease activity, efficacy, safety

## Abstract

**Background:**

Etanercept biosimilar recombinant human TNF-α receptor II: IgG Fc fusion protein (rhTNFR-Fc) has showed its efficacy and safety in Chinese patients with rheumatoid arthritis. However, data on rhTNFR-Fc's application in juvenile idiopathic arthritis (JIA) is limited.

**Methods:**

A prospective, observational, multicenter study was performed at 6 institutes in China from July 2020 to December 2021. In a 24-week follow-up, patients with JIA including polyarticular JIA and enthesitis related arthritis received rhTNFR-Fc plus methotrexate (MTX) treatment. The primary outcome parameters were improvements of cJADAS-10 (clinical Juvenile Arthritis Disease Activity Score), and the secondary outcome parameter was an inactive disease.

**Results:**

60 patients completed at least 12-week follow-up, and 57 completed 24-week follow-up. They had high C reactive protein values (11.6 mg/L) and cJADAS-10 (14.6) at baseline. Thirteen patients had morning stiffness. 33 patients showed synovial thickening, and 34 showed bone marrow edemas on MRI. Ultrasonography demonstrated significant joint effusions in 43 patients. The cJADAS-10 sharply decreased from 14.66 at the baseline to 2.4 at 24 weeks of rhTNFR-Fc therapy, respectively (*P* < 0.01). About half of patients achieved inactive disease at 24 weeks of therapy. Compared with the baseline, the number of patients with morning stiffness, joint effusions, bone marrow edema and synovial thickening on MRI significantly decreased at 24 weeks. Adverse events were consistent with known side effects of biologic agents.

**Conclusions:**

The present study indicated that the combination of rhTNFR-Fc and MTX significantly improve symptoms and disease activity of children with JIA. This study suggests etanercept biosimilar rhTNFR-Fc as an effective and safe therapy for children with JIA.

## Introduction

Juvenile idiopathic arthritis (JIA) is a heterogeneous collection of inflammatory arthritis of unknown etiology, and is also the most common chronic autoimmune disease in children (1, 2). According to International League of Associations for Rheumatology (ILAR) classification criteria, JIA is categorized into seven subtypes, including polyarticular course JIA (pJIA) and enthesitis related arthritis (ERA) (3, 4). The majority of children with JIA experienced continuously ongoing disease activity (5). Extended disease developed in one-third of the patients with oligoarticular JIA, and the outcomes of these patients were similar to those of children with pJIA (5). Furthermore, children with pJIA have a more treatment-resistant disease course than those with fewer joints affected, and they have longer periods of active disease associated with higher risk of joint damage (5, 6). Although some children with ERA appear to respond well to conventional synthetic disease-modifying anti-rheumatic drugs (cDMARDs) such as methotrexate or sulfasalazine monotherapy, most of children with peripheral joint disease still require the escalation of therapy to a biologic DMARDs (bDMARDs) (7, 8). Accordingly, recent treatment recommendations for pJIA or ERA suggest the bDMARDs as initial treatment or escalating therapy after 3–6 months' use of cDMARDs without response (3, 9). Tumor necrosis factor inhibitor (TNFi) has been recommended as a first-line biologic agent for the treatment of pJIA and ERA, including adalimumab and etanercept (3, 9).

Different from a TNF monoclonal antibody adalimumab, etanercept is a soluble receptor fusion protein consisting of the extracellular ligand-binding portions of human TNF p75 receptor, which binds and neutralize soluble and transmembrane TNF as well as lymphotoxin (10, 11). However, the high cost of etanercepts and the lack of national medical insurance coverage limited their use in China. The biosimilars of TNFi had an overall comparable efficacy and safety profile compared to their reference agents in rheumatoid arthritis and ankylosing spondylitis (12). Etanercept biosimilar recombinant human TNF-α receptor II: IgG Fc fusion protein (rhTNFR-Fc) has been widely used for more than 10 years as well as reduces the medical burden in China (13–17). rhTNFR-Fc has also showed its efficacy and safety in Chinese patients with rheumatoid arthritis, ankylosing spondylitis, and psoriasis (13, 14, 18, 19). Although rhTNFR-Fc has been approved and administered in Chinese children with pJIA and ERA, we lack a multi-center clinical study to further assess their efficacy and safety.

Here, we performed a prospective observational, open-label and multi-center study of rhTNFR-Fc in Chinese children with pJIA and ERA. The purpose of this study was to further evaluate the efficacy and safety of rhTNFR-Fc.

## Methods

### Study subjects

A prospective observational, open-label and multi-center study of etanercept biosimilar rhTNFR-Fc (trade name Yisaipu, 3SBio Inc., China) was carried out at 6 institutes (Zhejiang University School of Medicine Children's Hospital, Wenzhou Medical University Yuying Children's Hospital, Jiangxi Provincial Children's Hospital, Xiamen University Affiliated First Hospital, Soochow University Children's Hospital, Ningbo Women and Children's Hospital) in China from July 2020 to December 2021. The study protocol was conducted in accordance with the Declaration of Helsinki and Good Clinical Practice Guidelines and was approved by the ethics committee institutional review board (2020-IRB-096). This trial was registered in the Chinese Clinical Trial Registry database (ChiCTR2000035016).

All the children with JIA were followed up to assess the efficacy and safety of rhTNFR-Fc within 6 months of recruitment. Inclusion criteria were as follows: (1) age: 2–17 years old; (2) meet the classification criteria of pJIA and ERA by the ILAR (4); (3) children with pJIA and ERA were still moderately active after 3 months of methotrexate (MTX) or still low activity after 6 months of MTX; (4) MTX medication needs to meet one of the following conditions: MTX-free time before taking MTX or baseline visit ≥4 weeks; MTX medication time before baseline ≥12 weeks, and stable dose (10–15 mg/m^2^) for 8 weeks, and taking folic acid; (5) children who did not take oral corticosteroids, or the hormones have been discontinued for 4 weeks; (6) Children who did not receive non-steroidal anti-inflammatory drugs (NSAIDs); or are taking one kind of NSAIDs, but the stable dose should be applied for ≥2 weeks before the baseline visit. Exclusion criteria were as follows: (1) suffering from other autoimmune or rheumatic diseases other than JIA; (2) accompanied by serious infectious diseases, including but not limited to active tuberculosis, latent tuberculosis infection, active viral hepatitis; (3) severe gastrointestinal disease or previous medical history, such as ulcer, perforation or inflammatory bowel disease, Crohn's disease, ulcerative colitis; (4) a history of macrophage activation syndrome within 3 months before the screening visit; (5) previous history of demyelinating syndrome or multiple sclerosis; (6) received intra-articular, intramuscular, intravenous, or long-acting glucocorticoids (CS) treatment within 28 days before the baseline visit; (7) received other cDMARDs (except MTX) within 6 weeks before the baseline visit; (8) received cyclophosphamide treatment within 90 days before the baseline visit; (9) received live or attenuated vaccination within 4 weeks before the baseline visit, or plan to receive live or attenuated vaccination during study drug administration; (10) peripheral blood leukocyte count <4.0 × 10^9^/L, or peripheral blood neutrophil count <1.5 × 10^9^/L, or peripheral blood platelet count <100 × 10^9^/L, or serum creatinine >1.5 times the upper limit of reference value, or serum ALT >2 times the upper limit of reference value. The patient's clinical information including age, gender, laboratory tests, magnetic resonance imaging (MRI) and ultrasonography results were recorded.

### Drug administration

At treatment initiation, children with JIA received rhTNFR-Fc at a dose of 0.8 mg/kg per week (up to 50 mg/week) plus MTX at a dose of 10 mg/m^2^/week by subcutaneous injection and orally, respectively. The patients also received oral once-weekly folic acid on the second day of oral methotrexate and oral daily non-steroidal anti-inflammatory drugs (NSAIDs). When symptoms improved, NSAIDs were tapered. The therapeutic course was 24 weeks.

### Outcome criteria

The primary outcome parameters of the study were improvements of cJADAS-10. The cJADAS-10 was calculated by assessing the following variables: (1) physician's global rating of overall disease activity measured on a 0–10 visual analog scale (VAS), where 0 = no activity and 10 = maximum activity; (2) parent/child ratings of well-being assessed on a 0–10 VAS, where 0 = best and 10 = worst; and 3) counts of active joints assessed in 10 joints (the number of active joints of included patients <10) (20). The secondary outcome parameter was an inactive disease. The definition of inactive disease included no active uveitis or arthritis; no fever, rash, splenomegaly, serositis, generalized lymphadenopathy or elevation of ESR/CRP (erythrocyte sedimentation rate/C reactive protein); best physician's VAS; and duration of morning stiffness of ≤15 min (21). The cJADAS-10 cutoff value ≤1 was considered as inactive disease for pJIA and ERA (20). All the clinical assessments were performed at baseline and at week 4, 8, 12, and 24 after study initiation.

### Safety assessment

Safety was evaluated according to the frequency of adverse events (AEs) and laboratory abnormality. AEs were recorded in detail including the date and time of onset, description, severity, time course, duration and outcome, and relationship of the AE to the study drug. Severe AEs were defined as events that were fatal or life threatening and resulted in a persistent or major disability or incapacity requiring prolonged inpatient hospitalization (22).

### Statistical analysis

All the statistical analysis and graphics were performed with R statistical software packages (R version 4.0.3). Statistical analysis was performed using descriptive statistics. The continuous variables between groups were compared by Student's *t*-test or Mann-Whitney *U* test. For categorical variables, Pearson's chi-squared test was applied. A repeated measures analysis of variance was performed between children with pJIA and ERA. A *P* value of less than 0.05 was considered statistically significant.

## Results

### Characteristics of included children

**Figure 1:**
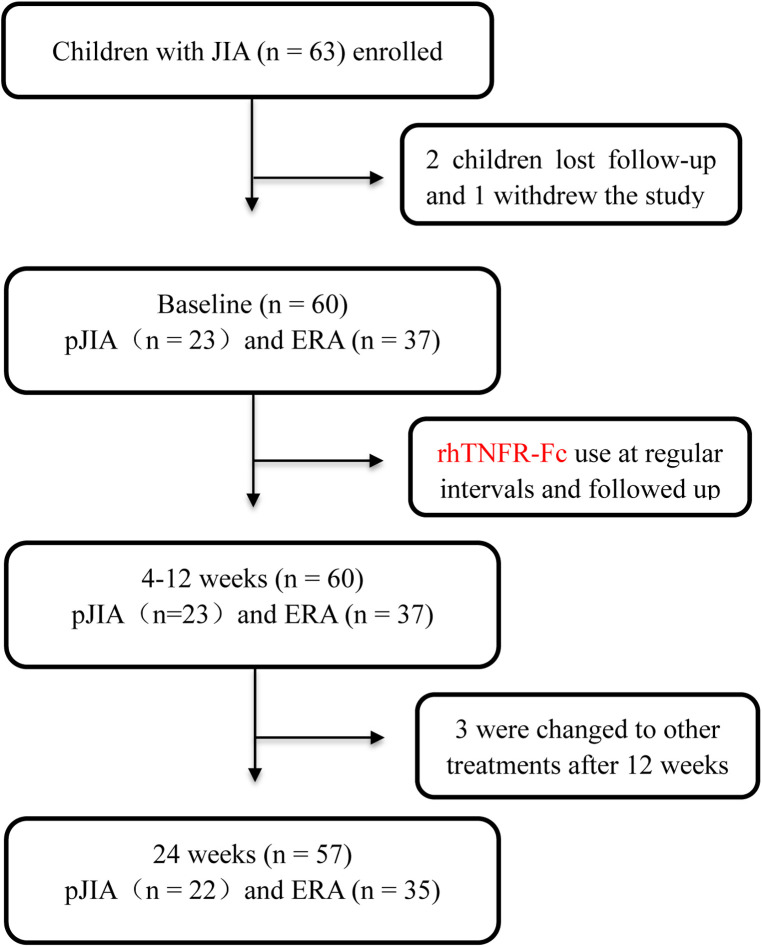
Participant selection flowchart. JIA, juvenile idiopathic arthritis; pJIA, polyarticular course JIA; ERA, enthesitis related arthritis.

The flow chart showed information for patient screening (Figure 1). 60 patients completed at least 12-week follow-up, and 57 completed 24-week follow-up. Detailed baseline clinical characteristics of children with JIA were listed in Table 1. These children included 30 boys and 30 girls, and their mean age was 8.6 ± 3.6 years with an average duration of symptoms of seven months. They had high CRP values (11.6 ± 17.5 mg/L) and cJADAS-10 (14.6 ± 5.7). Thirteen patients had morning stiffness; 33 patients showed synovial thickening, 34 showed bone marrow edemas, and 27 had surrounding soft swelling on the MRI. Ultrasonography demonstrated significant joint effusions in 43 patients. In addition, two subgroups of pJIA and ERA were also analyzed. At baseline, no significant differences were observed in age, duration of symptom, WBC (white blood cell) counts, platelets (PLT) counts, ESR, CRP, alanine transaminase (ALT), and creatinine (CREA) between the pJIA and ERA groups. Overall, there was a trend towards a higher cJADAS-10 with a more pronounced joint involvement in the pJIA group, as well as a significant female predominance.

**Table 1:** Clinical characteristics of children with JIA.

Clinical variables	Total, no. (%) (*n* = 60)	ERA, no. (%) (*n* = 37)	pJIA, no. (%) (*n* = 23)	*P* value
**Demographics**				
Age (years), mean (SD)	8.6 (3.9)	9.2 (3.8)	7.6 (3.9)	0.122
Male/female (male%)	30/30 (50%)	26/11 (70.3)	4/19 (17.4)	<0.01**
Height, mean (SD), cm	133 (24.9)	138.3 (23.9)	124.6 (24.7)	0.037*
Weight, median (IQR), kg	27.9 (18.5-38.9)	30 (23.5-42.4)	25 (15.5-36.8)	0.092
**Disease characteristics**				
Symptom duration, Median (IQR), months	7 (4-14.25)	8 (4-14)	6 (4.5-14)	0.882
WBC				
Median (IQR), 10^9^/L	7.61 (6.64-8.60)	7.60 (6.81-8.84)	7.67 (6.31-8.17)	0.68
In reference range	54 (90.0)	33 (89.2)	21 (91.3)	0.79
Outside reference range	6 (10.0)	4 (10.8)	2 (8.7)	
Hgb				
Mean (SD), g/L	120.3 (15.5)	124.2 (14.4)	113.8 (15.6)	0.015*
In reference range	58 (96.7)	36 (97.3)	22 (95.7)	0.73
Outside reference range	2 (3.3)	1 (2.7)	1 (4.3)	
PLT				
Mean (SD), 10^9^/L	372.6 (11.5)	375.7 (11.7)	367.6 (20.5)	0.73
In reference range	31 (68.3)	25 (67.6)	16 (69.6)	0.87
Outside reference range	19 (31.7)	12 (32.4)	7 (30.4)	
ESR				
Median (IQR), mm/h	20 (10.5-41.5)	19 (12-38)	22 (9-44.5)	0.486
In reference range	31 (51.7)	20 (54.1)	11 (47.9)	0.16
Outside reference range	29 (48.3)	17 (45.9)	12 (52.1)	
CRP				
Mean (SD), mg/L	11.6 (17.5)	9.0 (13.0)	15.9 (22.6)	0.19
In reference range	39 (65.0)	25 (67.6)	14 (60.9)	0.60
Outside reference range	21 (35.0)	12 (32.4)	9 (39.1)	
ALT				
Mean (SD), IU/L	17.3 (11.7)	19 (12.9)	14.5 (9.1)	0.118
In reference range	59 (98.3)	36 (97.3)	23 (100)	0.43
Outside reference range	1 (1.7)	1 (2.7)	0 (0)	
CREA				
Mean (SD), µmol/L	48.7 (7.23)	53.8 (11.4)	40.6 (4.1)	0.38
In reference range	59 (98.3)	36 (97.3)	23 (100)	0.43
Outside reference range	1 (1.7)	1 (2.7)	0 (0)	
Physician's global rating, Mean (SD)	5.3 (2.2)	5.1 (2.2)	5.6 (2.2)	0.40
Parent/child rating, mean (SD)	5.5 (2.4)	5.5 (2.5)	5.5 (2.3)	0.96
Counts of active joints, Mean (SD)	4.1 (3.5)	2.4 (1.5)	6.9 (3.9)	< 0.01**
cJADAs				
Mean (SD)	14.6 (5.7)	13 (5)	17.3 (5.8)	< 0.01**
≤2.5	0 (0)	0 (0)	0 (0)	
>2.5 / ≤8.5	9 (15.0)	7 (18.9)	2 (8.7)	0.28
>8.5	51 (85.0)	30 (81.1)	21 (91.3)	
Morning stiffness	13 (21.7)	4 (10.8)	9 (39.1)	< 0.01**
MRI synovial thickening	33 (55)	23 (62.2)	10 (43.5)	0.1572
MRI: bone marrow edema	34 (56.7)	19 (51.4)	15 (65.2)	0.292
MRI: peripheral soft tissue swelling	27 (45)	16 (43.2)	11 (47.8)	0.7286
Ultrasound joint effusions	43 (71.7)	25 (67.6)	18 (78.3)	0.3715

### Efficacy

**Figure 2:**
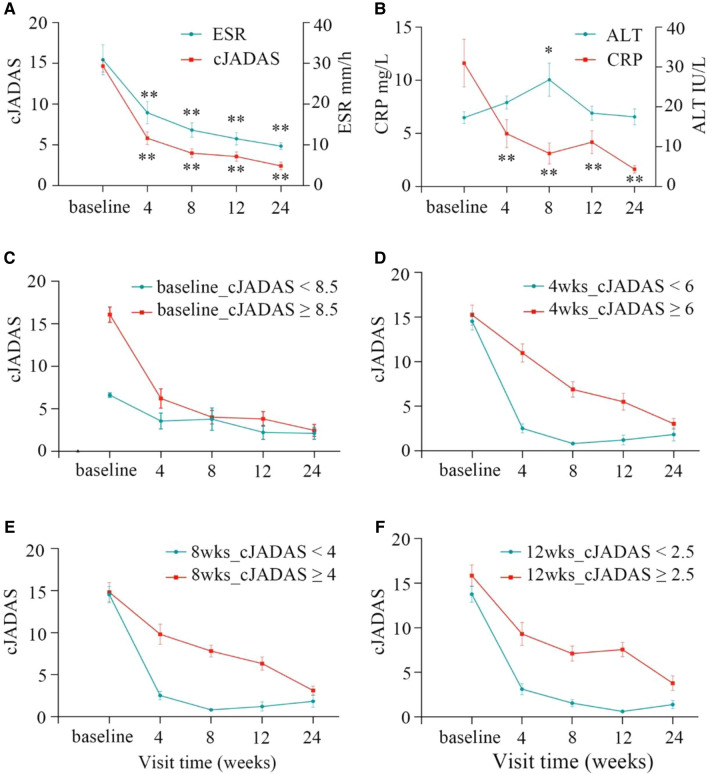
Clinical response at baseline and after 4, 8, 12, 24 weeks of rhTNFR-Fc therapy. (**A**) The cJADAS-10 sharply decreased from 14.66 at the baseline to 5.81 at 4 weeks (*P* < 0.001), and the cJADAS-10 decline slowly from 3.97 to 2.40 at 8 weeks and 24 weeks; the similar trend exists in ESR. (**B**) The decreased trend was observed in CRP; ALT were within normal ranges with moderately elevation at week 8. The similar trend to the total cJADAS-10 (**A**) was showed in various cJADAS-10 subgroups based on the baseline (**C**), and mean cJADAS-10 of 4 (**D**), 8 (**E**), and 12 (**F**) weeks after treatment (*P* < 0.01). cJADAS, clinical Juvenile Arthritis Disease Activity Score; ESR, erythrocyte sedimentation rate; CRP, C reactive protein.

The significant improvements were observed from the baseline to the last visit in the majority of patients. During the follow-up, three children withdrew this study after 12 weeks due to poor efficacy. Overall, these patients demonstrated clinical benefits by the decrease in cJADAS-10 (*P* < 0.01), erythrocyte sedimentation rate (ESR, *P* < 0.01), and C reactive protein (CRP, *P* < 0.01) throughout the follow-up period (Figures 2A,B). At 4 weeks, the three indicators showed a clear downward trend, especially cJADAS-10. Notably, the cJADAS-10 sharply decreased from 14.66 to 5.81 at the baseline and 4 weeks, respectively (*P* < 0.01). However, the cJADAS-10 declined slowly from 3.97 at 8 weeks to 2.40 at 24 weeks. Additionally, the cJADAS-10 subgroups were set up based on high disease activity and mean of cJADAS-10 at 4, 8, and 12 weeks after treatment (Figures 2C–F, Table 2). The repeated measures analysis of variance revealed that there were significant statistical differences between subgroups (*P* < 0.001). Similar to total cJADAS-10 trend (Figure 2A), various cJADAS-10 subgroups also demonstrated a clear decreasing trend from baseline to 4 weeks (Figures 2C–F). Furthermore, the number of children with inactive disease of cJADAS-10 ≤ 1 significantly increased from zero at baseline to 19 at 4 weeks (Table 2), indicating that 4 weeks of rhTNFR-Fc treatment contributed to significant improvements in the cJADAS-10 and clinical manifestations.

**Table 2:** Clinical characteristics of various cJADAS-10 subgroups.

cJADAS-10 subgroups	Baseline (*n* = 60)	4 weeks (*n* = 60)	8 weeks (*n* = 60)	12 weeks (*n* = 60)	24 weeks (*n* = 57)
cJADAs ≤ 1, no. (%)	0 (0)	19 (31.67)	22 (36.67)	26 (43.33)	29 (53.70)
Total cJADAs, mean (SD)	14.66 (5.71)	5.81 (5.93)	3.97 (4.34)	3.56 (4.42)	2.40 (3.59)
**Subgroup based on baseline data**					
cJADAs <8.5, no. (%)	9.00 (15.00)	9.00 (15.00)	9.00 (15.00)	9.00 (15.00)	9.00 (15.79)
cJADAs <8.5, mean (SD)	6.61 (0.78)	3.56 (2.79)	3.78 (3.96)	2.22 (2.49)	2.11 (2.09)
cJADAs ≥8.5, mean (SD)	16.08 (4.96)	6.21 (6.25)	4.00 (4.44)	3.80 (4.66)	2.46 (3.83)
**Subgroup based on 4-week data**					
cJADAs <6, no. (%)	34.00 (56.67)	34.00 (56.67)	34.00 (56.67)	34.00 (56.67)	33.00 (57.89)
cJADAs <6, mean (SD)	14.22 (5.65)	1.85 (1.97)	1.74 (2.50)	2.03 (3.35)	1.94 (3.87)
cJADAs ≥6, mean (SD)	15.23 (5.85)	10.98 (5.35)	6.88 (4.53)	5.50 (4.89)	3.04 (3.14)
**Subgroup based on 8-week data**					
cJADAs <4, no. (%)	33.00 (55.00)	33.00 (55.00)	33.00 (55.00)	33.00 (55.00)	32.00 (56.14)
cJADAs <4, mean (SD)	14.53 (5.55)	2.53 (2.82)	0.82 (1.10)	1.22 (3.14)	1.84 (4.06)
cJADAs ≥4, mean (SD)	14.81 (5.99)	9.81 (6.29)	7.81 (3.64)	6.33 (4.13)	3.12 (2.80)
**Subgroup based on 12-week data**					
cJADAs <2.5, no. (%)	34 (56.67)	34 (56.67)	34 (56.67)	34 (56.67)	33 (57.89)
cJADAs <2.5, mean (SD)	13.75 (5.17)	3.13 (3.52)	1.56 (2.30)	0.62 (0.85)	1.39 (2.65)
cJADAs ≥2.5, mean (SD)	15.85 (6.25)	9.31 (6.64)	7.12 (4.38)	7.56 (4.15)	3.79 (4.27)

**Figure 3:**
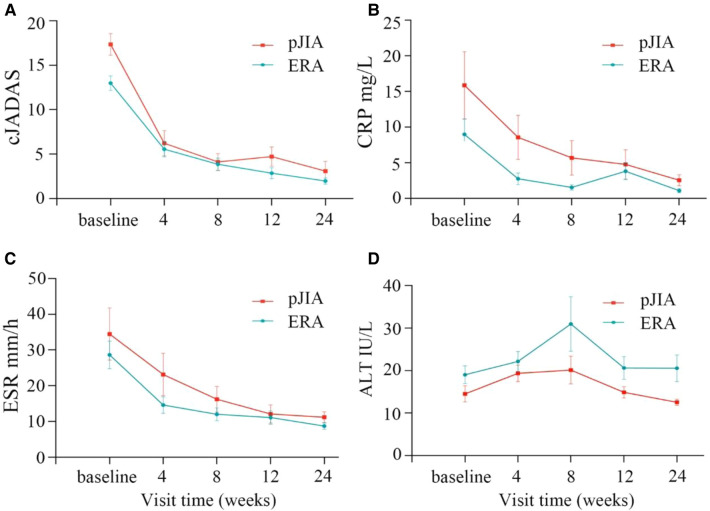
Comparison of children with pJIA and ERA. There were obvious decreased trends in cJADAS-10 (**A**), CRP (**B**) and ESR (**C**) between children with pJIA and ERA. The repeated measures analysis of variance revealed significant statistical differences in the cJADAS-10 (**A**), CRP (**B**), and ESR (**C**) between the two groups. The ALT levels were still within normal range regardless of pJIA or ERA (**D**). pJIA, polyarticular course JIA; ERA, enthesitis related arthritis; cJADAS, clinical Juvenile Arthritis Disease Activity Score; ESR, erythrocyte sedimentation rate; CRP, C reactive protein.

The subgroup analysis showed that patients with pJIA had higher cJADAS-10 score, CRP and ESR values compared with those with ERA (Figure 3). The repeated measures analysis of variance revealed that there were significant statistical differences in the cJADAS-10 (*P* = 0.023), CRP (*P* < 0.001), and ESR (*P* = 0.001) between the two groups (Figures 3A–C). Consistent with total cJADAS-10 and cJADAS-10 subgroups, 4 weeks of rhTNFR-Fc treatment also contributed to a significant improvement in the cJADAS-10 regardless of pJIA or ERA, as well as in CRP and ESR.

**Figure 4:**
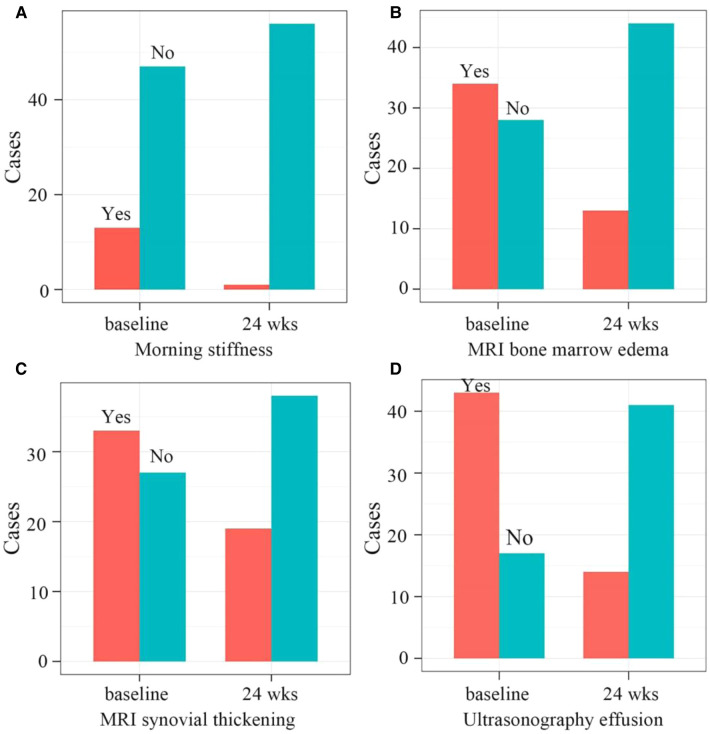
(**A**) the number of patients with morning stiffness significantly decreased from 13 to 1 at the baseline and 24 weeks after therapy, respectively (*P* < 0.001). (**B**) The number of patients with bone marrow edema on MRI significantly decreased from 34 to 13 at the baseline and 24 weeks after therapy, respectively (*P* < 0.001). (**C**) the number of patients with synovial thickening on MRI significantly decreased from 33 to 19 at the baseline and 24 weeks after therapy, respectively (*P* = 0.0184). (**D**) The number of patients with ultrasonography effusions significantly decreased from 43 to 14 at the baseline and 24 weeks after therapy, respectively (*P* < 0.001). MRI, Magnetic Resonance Imaging.

Compared with the baseline, the number of patients with morning stiffness (*P* < 0.001) and joint effusions (ultrasonography, *P* < 0.001) significantly decreased at 24 weeks. Furthermore, the number of children with bone marrow edema (*P* < 0.001) and synovial thickening (*P* = 0.018) on MRI also significantly decreased at 24 weeks (Figure 4).

### Safety

Treatment safety was evaluated separately at each visit, including complete blood counts, liver and kidney functions. At the last visit (24 weeks), infections including hepatitis virus and tuberculosis were detected. AEs and severe AEs were also recorded at each visit. Overall, both MTX and rhTNFR-Fc were well tolerated, and the majority of AEs were mild or moderate. During the 24-week follow-up period, a transient decrease of leucocyte counts (<4 × 10^9^/L) in 4 patients, an elevation of liver enzyme (ALT > 40 IU/L) in 4 patients and increased uric acid levels (range 420–639 µmol/L) in 8 patients were observed. Although a transient elevation of liver enzyme was observed at 8 weeks after therapy, their levels were still within normal range (Figures 2B, 3D), indicating the importance of liver function monitoring, especially within 8 weeks after therapy.

AEs were consistent with known side effects of biologic agents, including transient reaction of the injection site, skin rash, and gastrointestinal symptom. Upper respiratory tract infections occurred in seven patients. There were no infections requiring hospitalization or intravenous administration of antibiotics. AEs were mild, not requiring interruption of the rhTNFR-Fc, DMARDs, and NSAIDs therapy. No severe AEs and deaths occurred during the course of our study. One patient had a positive PPD test and received prophylactic antituberculosis drugs.

## Discussion

Tumor necrosis factor-α (TNF-α) is a naturally occurring cytokine that produced by T-cells and macrophages, integrally involving in both the physiologic and pathologic processes of the inflammatory and immune responses (23). Many biologic agents that target against TNF-a have been developed and are now increasingly used in clinical practice (1, 2). TNFi is effective against disease activity and improves the physical functionality of patients with ankylosing spondylitis. Furthermore, TNFi may retard the progression of spinal mobility dysfunction and maintain spinal mobility (14). Several biosimilars of TNFi have been approved and marketed in various countries. Furthermore, TNFi biosimilars had been shown to have an overall comparable efficacy and safety profile compared to their reference agents in rheumatoid arthritis and ankylosing spondylitis (12). Etanercept is a fusion protein consisting of a Fc portion of immunoglobulin G linked to human TNF-α receptor with potent anti-inflammatory activities, competitively inhibiting the binding of TNF-α to cell surface receptors and attenuating its biological effects. A number of studies have revealed the good effect and safety of etanercept for the treatment of rheumatoid arthritis, JIA, psoriatic arthritis, ankylosing spondylitis and psoriasis (10, 24–27).

Etanercept biosimilar recombinant human TNF-α receptor II: IgG Fc fusion protein (rhTNFR-Fc) is a TNF-α inhibitor targeting soluble TNF-α to inhibit its interaction with cell–surface receptors. Currently, rhTNFR-Fc has been widely used in clinical practice for more than 10 years in China (15, 17, 19). The efficacy and safety of rhTNFR-Fc have been also confirmed in Chinese patients with rheumatoid arthritis, plaque psoriasis, ankylosing spondylitis, and axial spondyloarthritis (13, 14, 16–19). Our study also showed that the combination of rhTNFR-Fc and MTX significantly improve symptoms of children with pJIA and ERA, and nearly half of patients achieved inactive disease at 24 weeks of therapy. This study further supported the role of rhTNFR-Fc in immune-mediated inflammatory conditions, regardless of adult rheumatoid arthritis or JIA. A randomized, double-blind multicenter study reported that adalimumab was shown to reduce signs and symptoms of ERA at week 12 (28). Our multi-center study demonstrated that a significant improvement in cJADAS-10 was observed at the fourth week of rhTNFR-Fc therapy, suggesting an available early time window suitable for observing efficacy. Although three patients failed to respond to 12-week treatment and switched to other treatments, most of patients achieved better clinical improvement such as morning stiffness, bone marrow edema, synovial thickening, and joint effusions, further supporting an efficacy of rhTNFR-Fc.

Compared to those treated with the standard step-up regimen, more patients treated with a combination of etanercept plus MTX reached better response and achieved inactive disease and remission more rapidly (26). The majority of children with JIA can attain inactive disease within 2 years, even many being able to discontinue treatment (6). In general, patients with JIA did not used biological DMARDs until they were treated with cDMARD at adequate dose for at least 3 months (3, 9). However, such a recommendation would prolong the time with active arthritis, reduce the life quality of patients and increase the risk of developing irreversible osteoarticular changes (26). According to 2019 Arthritis Foundation Guideline, initial biologic therapy may be considered for patients with risk factors and involvement of high-risk joints, high disease activity, and/or those judged by their physician to be at high risk of disabling joint damage (9). Furthermore, most patients have concerns about corticosteroids side effects, they prefer biologics as the first-line choice. In the present study, some patients directly chose biologic agents as their initial treatment due to high-risk factors. Different from a longer duration in adult rheumatic diseases, the duration of JIA in our study was relatively short. We observed a high response rate, with 50% of patients with JIA achieving inactive disease after 24 weeks of rhTNFR-Fc treatment. These results allowed us to speculate that early biologic agents or combination treatment could contribute to improvement of symptoms and control of disease. On the other hand, the safe use of biosimilars depends on informed and adequate administration by medical professionals and regulatory agencies (29). In the field of pediatric rheumatology, further education about biosimilars and real-life experiences is required to better understand treatment options in children (30). There is evidence that adalimumab biosimilar is a suitable and effective treatment option for patients with JIA and has a gradual increase in prescription in pediatric rheumatology (31), which further indicating that these biosimilars had similar efficacies to reference agents.

Notably, cJADAS-10 was frequently used to assess the condition of pJIA patients (20). However, for ERA, there are currently no widely accepted scoring criteria. In the view of convenience and operability of cJADAS-10, we adopted cJADAS-10 as scoring criteria for pJIA and ERA. Especially in the multi-center study, consistency is very important for different hospitals. Our study indicated that cJADAS-10 was also feasible in evaluating ERA.

Regarding the safety assessment, we found that the combination regime had an acceptable safety and tolerability profile. AEs were consistent with known side effects of biologic agents, including transient reaction of the injection site, skin rash, and gastrointestinal symptom. Additionally, the most common AEs were upper respiratory tract infections, elevation of liver enzyme, and increased uric acid levels. Overall, AEs were mild or moderate, not requiring interruption of the rhTNFR-Fc therapy. No severe AEs and deaths were observed during the course of our study. Notably, a transient elevation of liver enzyme was observed at 8 weeks after therapy, suggesting the importance of liver function monitoring within 8 weeks after therapy. In addition to adult rheumatic diseases, our study also supported the safety of rhTNFR-Fc in patients with JIA.

Additionally, we also need to focus on the nocebo effect of biosimilars. Nocebo effects were considered as new or worsening symptoms or adverse events that occur largely as a consequence of patients' negative expectations rather than by the mechanism of the treatment itself (32). The nocebo effect could further inhibit biosimilar adoption, especially those patients previously discomfort with switching to a biosimilar product (31). In this study, the enrolled patients were the first to use biologics, we did not find significant nocebo effect. There was evidence that systematic switch from reference to biosimilar etanercept was not associated with changes in disease activity or function (33). Current evidence is insufficient to confirm a biosimilar nocebo effect (34).

The present study has some limitations that should be considered. First, our study did not use the control group and double-blind method. Second, this study enrolled Chinese children and no other ethnicities were included. Therefore, the present findings may not be completely applicable to other ethnicities. Third, the sample size was relatively small due to difficulty in recruiting pediatric patients. Although our results are derived from only 24-week follow-up records, this can still reflect the true efficacy and safety of rhTNFR-Fc. In addition, we are also performing a longer follow-up to further make a more comprehensive assessment of rhTNFR-Fc's efficacy and safety.

## Conclusion

This prospective, observational, open-label and multi-center study indicated that the combination of rhTNFR-Fc and MTX significantly improve symptoms and disease activity of children with pJIA and ERA. Furthermore, nearly half of patients achieved inactive disease at the end of follow-up. Throughout the follow-up, AEs were consistent with known side effects of biologic agents. These findings suggest rhTNFR-Fc as an effective and safe therapy for children with pJIA and ERA, especially for those patients with involvement of high-risk joints (cervical spine, wrist, or hip) and/or high disease activity. Further multicenter randomized controlled trial should be conducted on rhTNFR-Fc in patients with JIA or other rheumatic diseases.

## Data Availability

The raw data supporting the conclusions of this article will be made available by the authors, without undue reservation.
